# Deep-learning based image reconstruction enables reduced dose CT pulmonary angiography with non-inferior image quality

**DOI:** 10.1038/s41598-026-56545-y

**Published:** 2026-06-09

**Authors:** Sina Sender, Paul J. Böttcher, Ann-Christin Klemenz, Tobias Geyer, Justus Hillebrand, Matthias Lütgens, Roberto Lorbeer, Mathias Manzke, Marc-André Weber, Felix G. Meinel

**Affiliations:** 1 Institute and Policlinic of Radiology, Pediatric Radiology and Neuroradiology, University Medical Centre Rostock, Schillingallee 36, 18057 Rostock, Germany; 2 Department of Radiology, Ludwig-Maximilian University, Munich, Germany

**Keywords:** Thorax, Pulmonary embolism, Computed tomography angiography, Deep learning, Radiation protection, Diseases, Health care, Medical research

## Abstract

To investigate whether a CT pulmonary angiography (CTPA) protocol with reduced radiation dose and deep-learning based image reconstruction (DLIR) is non-inferior in image quality to standard-dose CTPA using iterative reconstruction. A phantom study was conducted to estimate the additional radiation dose reduction enabled by high-strength deep learning-based image reconstruction (DLIR-H) compared to adaptive statistical iterative reconstruction (ASiR-V 90%). Medium and large phantoms were used to simulate different body sizes. Subsequently, we reduced radiation dose of our clinical CTPA protocol and transitioned to DLIR for image reconstruction. We retrospectively analyzed 307 consecutive patients who were examined before (n = 152) and after (n = 155) this clinically driven change in the CTPA protocol. Objective image quality was quantified and subjective image quality was rated by two radiologists. The non-inferiority margin was pre-specified as a < 5% difference in image quality parameters. In the phantom, DLIR-H allowed radiation dose to be reduced by up to 71% with equivalent or higher signal-to-noise-ratio (SNR) compared to standard-dose examinations reconstructed with ASiR-V 90%. In the patient cohort, radiation dose was reduced by 41% (median DLP 116 vs. 68 mGy*cm; effective dose 1.69 vs. 0.99 mSv, p < 0.001). In the modified protocol, median SNR was superior for the central pulmonary artery (13.6 vs. 22.3) and non-inferior for the segmental pulmonary arteries (16.4 vs. 16.8). Subjective image quality averaged over both readers was superior with the modified protocol. Compared to state-of-the-art iterative reconstruction, DLIR allows radiation dose for CTPA to be reduced by an additional 41% with non-inferior image quality.

**Supplementary Information:**

The online version contains supplementary material available at 10.1038/s41598-026-56545-y.

## Introduction

CT pulmonary angiography (CTPA) is the most commonly used imaging modality in the diagnosis of suspected pulmonary embolism (PE). Technical advances have substantially reduced the radiation dose associated with CTPA^1–3^. Some of these have revolved around improving the dose-efficiency of the CT hardware and acquisition process. These include reduced tube voltage scanning^4^, individualized tube voltage selection^5^ and tube current modulation, high-pitch scanning^6,7^ and – more recently – the development of photon-counting CT systems^7,8^.

Other approaches have focused on improving image reconstruction since powerful image reconstruction algorithms can maintain image quality with lower radiation dose. Various types and generations of iterative reconstruction (IR) algorithms have been developed and the contribution of IR in reducing the dose requirement for CTPA is well documented^9–14^. A more recent innovation in CT reconstruction technique is deep learning-based image reconstruction (DLIR). Using convolutional neural networks to reconstruct high-quality images from noisy raw data, DLIR algorithms can improve image quality by reducing image noise and artifacts beyond the capabilities of IR^15–17^.

Data concerning the application of DLIR in CPTA is very sparse. An intra-individual comparison of DLIR vs. IR reconstructions of identical CTPA studies in suspected PE has demonstrated that DLIR outperformed iterative reconstruction in both objective and subjective image quality^18^. This suggests that implantation of DLIR may allow for a further radiation dose reduction in CTPA. There is only one previous study directly showing that DLIR significantly improved image quality while also reducing radiation dose compared to IR for CTPA in suspected PE^19^.

Based on these previous studies and our own phantom experiments, we made the clinical decision in August 2024 to modify our routine CTPA protocol to use DLIR for image reconstruction and simultaneously decrease the radiation dose. In this retrospective study we aimed to investigate whether the modified CTPA protocol with reduced dose and high-strength DLIR (DLIR-H) is non-inferior in image quality for suspected PE compared to the original protocol using standard dose and high-strength iterative reconstruction (ASiR-V 90%).

## Results

### Phantom study

#### Impact of reference noise index on radiation dose

**Fig. 1:**
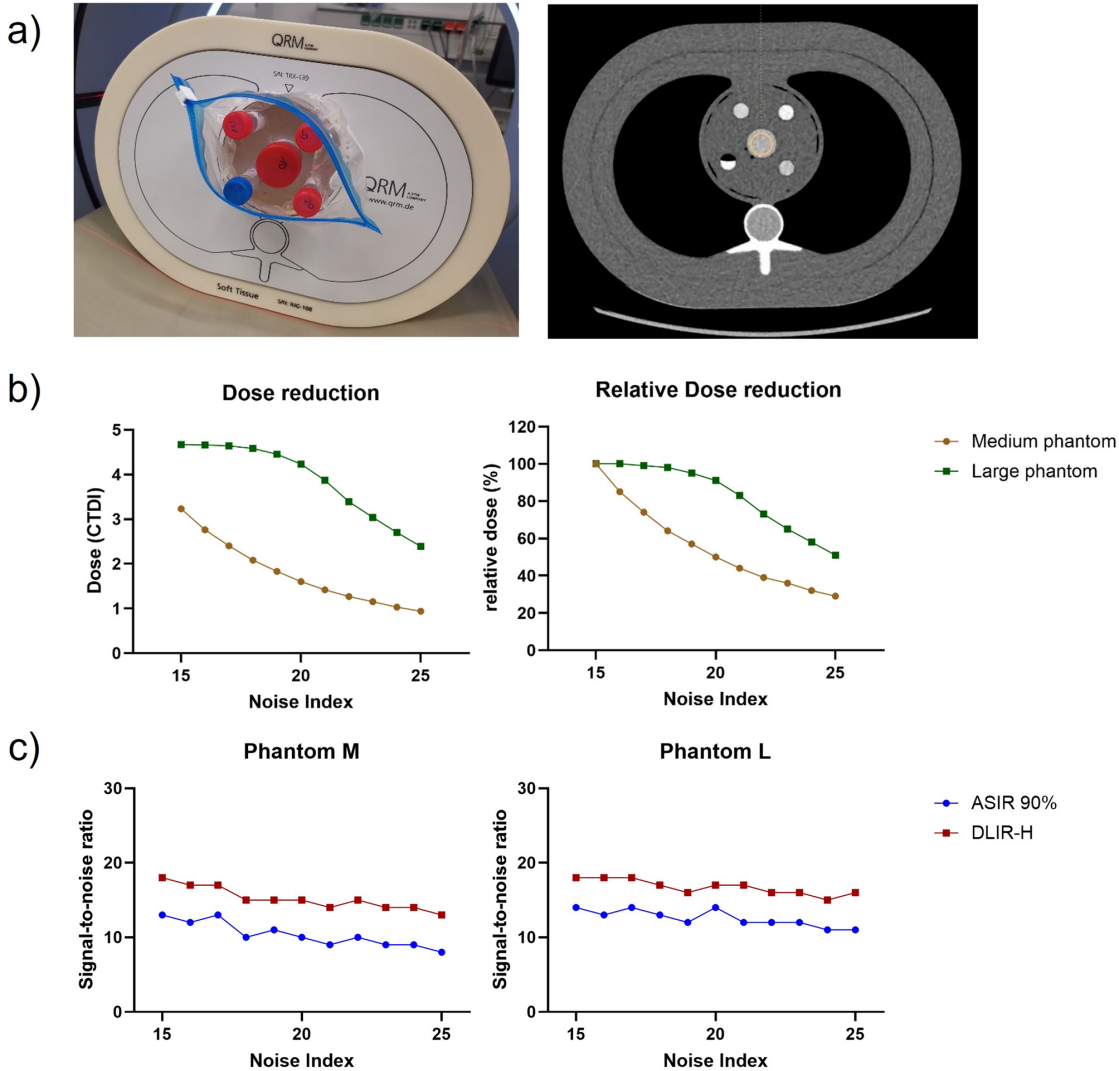
Thorax phantom setup and impact of dose reduction. (**a**) Front view of the thorax phantom setup equipped with 15 ml and 50 ml Falcon tubes in an agar-agar inlay (left) and CT scan of the equipped thorax phantom  (right). (**b**) The noise index was incrementally increased from 15 to 25 and dose for both phantoms was recorded. Dose (CTDI) decreases with increasing noise index for both phantoms. The relative dose (%) likewise decreases with increasing noise index. (**c**) Signal-to-noise ratio (SNR) decreased by increasing noise index, whereby the SNR was higher for DLIR-H reconstruction compared to ASiR-V 90%.

Incrementally increasing the noise index led to a decrease in radiation dose both for the medium phantom (simulating normal-weight individuals) and for the large phantom (simulating obese individuals), see Fig. 1b. For the medium phantom, radiation dose exponentially decreased with increasing noise index. For the large phantom, the dose reduction occurred only when the noise index was raised beyond 18. This was due to technical limitations of the tube current. To achieve a NI below 18 with the given rotation time (0.28 s) and fixed tube voltage (100 kVp), tube currents exceeding the maximum limit would be required. Consequently, the radiation dose remains nearly constant for NIs up to 18. Only with higher noise indices, radiation dose began to drop. Increasing the NI from 15 to 20 led to a 50% dose reduction for the medium phantom and a 9% dose reduction for the large phantom. Increasing the NI from 15 to 25 led to an 71% dose reduction for the medium phantom and a 49% dose reduction for the large phantom.

#### Impact of reference noise index on signal-to-noise ratio

Signal-to-noise ratio (SNR) decreased by increasing noise index for both phantoms and both reconstruction techniques (Fig. 1c). The SNR was significantly higher for DLIR-H compared to ASiR-V 90% for both phantoms. For the medium size phantom, the SNR for ASIR-V 90% was 13 for the original dose (noise index 15), the same level was reached with DLIR-H with a noise index of 25 (71% dose reduction). For the large phantom, the SNR for ASiR-V 90% was 14 for the original dose (NI 15). With DLIR-H, the SNR was higher (16) even with a noise index of 25 (49% dose reduction).

Based on these phantom results, we modified our clinical CTPA protocol to use DLIR-H and simultaneously lowered the dose by increasing the reference NI from 15 to 20. The new NI of 20 was chosen as a compromise achieving substantial dose reduction (50% for normal-weight individuals, less for obese patients) and caution to not risk any non-diagnostic examinations.

### Clinical study

#### Patient characteristics

A total of 307 patients (152 female, 155 male) with a median age of 68 years (range 18–96) were included into the study. 152 patients were examined with the original protocol and 155 patients with the modified protocol. No significant differences in patient characteristics were observed between both groups (Table 1).

**Table 1:** Patient characteristics.

Demographic parameters	All patients *n* = 307	Original protocol *n* = 152	Modified protocol *n* = 155	*p*-value
Age, median (range) in years	**68 ** **(18–96)**	69 (19–94)	67 (18–96)	0.44
Sex, number of women (%)	**152 (49.5%)**	79 (52.0%)	73 (47.1%)	0.39
Weight [kg]	**80** **(44–131)**	**80** **(44–128)**	**80** **(50–131)**	0.75
Height [m]	**1.70** **(1.47–2.10)**	**1.70** **(1.49–1.93)**	**1.72** **(1.47–2.10)**	0.29
BMI [kg/m2]	**26.7** **(15.5–48.5)**	**26.9** **(15.5–48.5)**	**26.6** **(18.1–45.6)**	0.68
Acute PE confirmed on CT	**67**	34	33	0.71

Complete data on weight and height for BMI calculation was available in 257 of 307 patients (129 patients in the original protocol and 128 patients in the modified protocol group). p-values are from Wilcoxon rank-sum (Mann–Whitney) test for non-normally distributed continuous data and chi-square test for categorical data.

#### Radiation dose

**Fig. 2:**
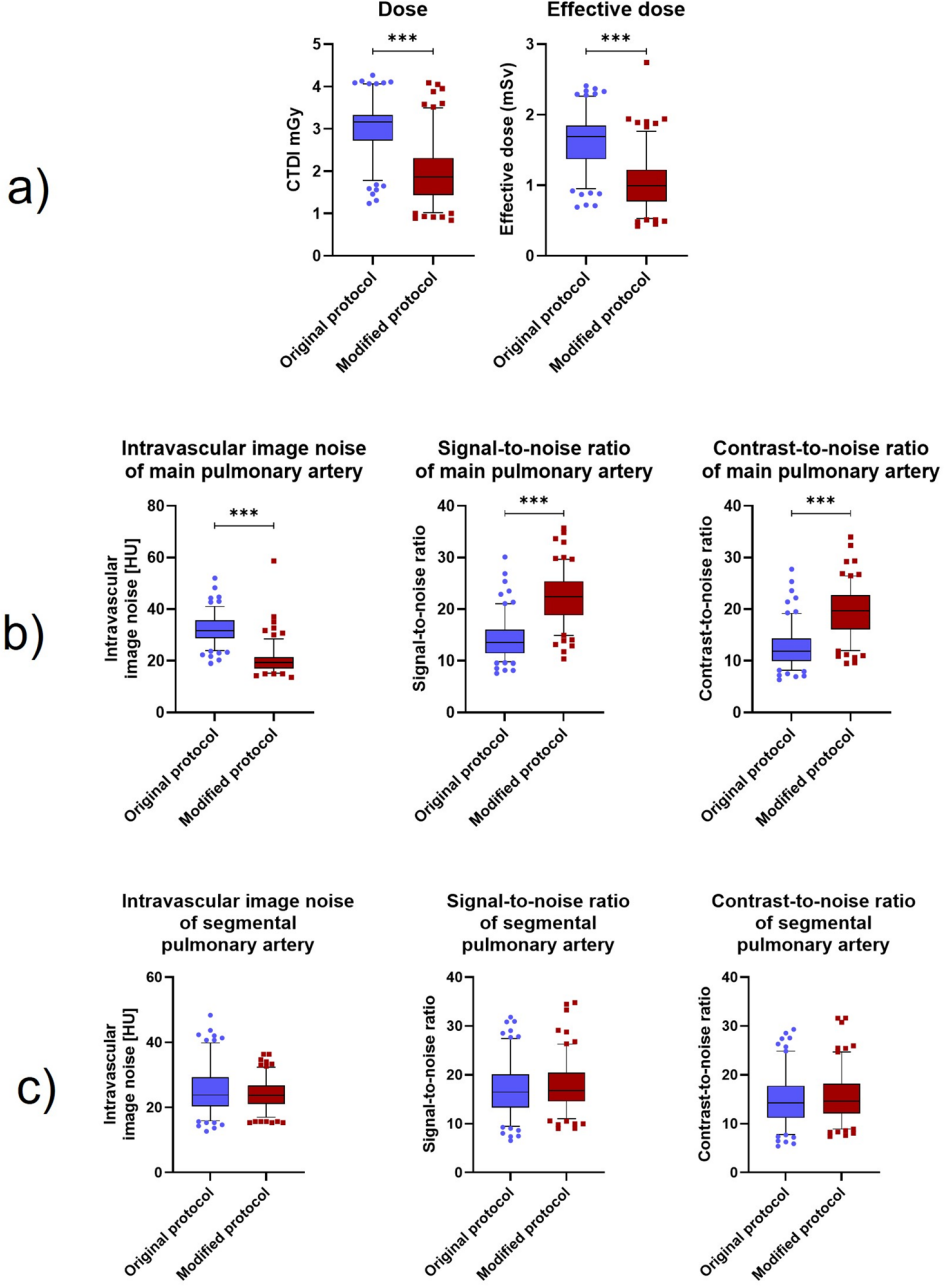
Radiation dose and objective image quality. Data are presented as median and 5th–95th percentile. Blue boxplots indicate the original protocol (standard dose and ASiR-V 90%) and red boxplots the modified protocol (reduced dose and DLIR-H). Statistical significance was calculated by Mann-Whitney test and displayed as *** *p* < 0.001.

Median radiation exposure was 41% lower with the modified protocol (median CTDI 3.17 vs. 1.86 mGy., median DLP 115.6 vs. 67.8 mGy*cm, *p* < 0.0001, Fig. 2a). Median effective dose was decreased below 1 mSv with the modified protocol (1.69 vs. 0.99 mSv, Fig. 2a).

#### Objective image quality

The results of the objective image quality analysis are summarized in supplementary Tables 4 and Fig. 2b and 2c. For the main pulmonary artery, median intravascular image noise was 39% lower in the modified protocol (median 31.7 vs. 19.3 HU, *p* < 0.001). This resulted in an 64% increase in SNR (13.6 vs. 22.3) and a 66% increase in CNR (11.8 vs. 19.6, both < 0.001). For the segmental pulmonary arteries, there were no significant differences between the modified and the original protocol regarding intravascular image noise, SNR and CNR.

**Fig. 3:**
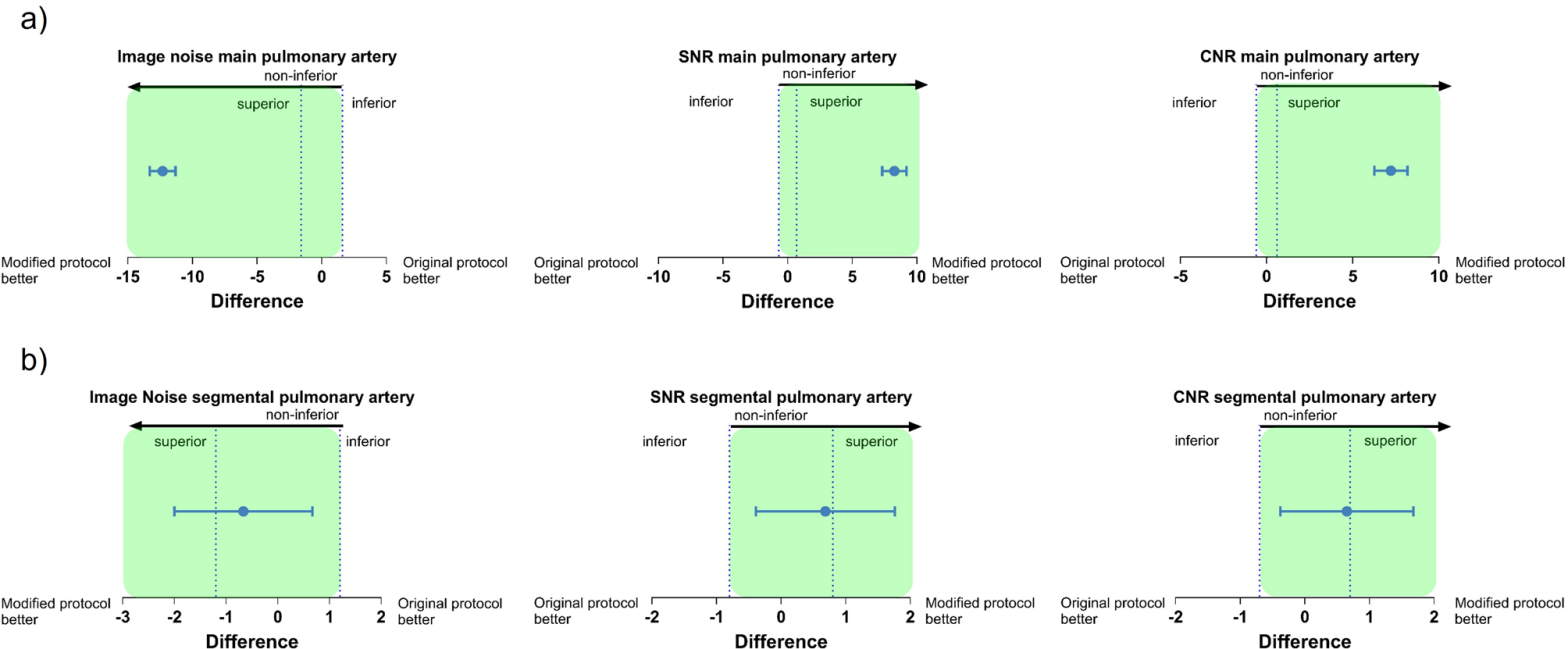
Non-inferiority analysis of objective image quality for the main and segmental pulmonary artery. Non-inferiority was evaluated based on image noise, SNR and CNR for (**a**) the main pulmonary artery and (**b**) the segmental pulmonary artery. Data are presented as median and 5th–95th percentile. Pre-defined non-inferiority as well as superior margins are marked as blue dashed line.

For the main pulmonary artery, the modified protocol was confirmed to be non-inferior and also superior to the original protocol regarding image noise, SNR and CNR (Fig. 3a). For the segmental pulmonary artery non-inferiority of the modified protocol was also confirmed but superiority was not seen (Fig. 3b).

#### Subjective image quality

Overall, the modified protocol received significantly higher image quality ratings compared to the original protocol from both readers (Table 2). The median image quality averaged over both readers was higher for the modified protocol both for the central and lobar pulmonary arteries (4 vs. 4.5, *p* < 0.001) and for the segmental and subsegmental pulmonary arteries (3.5 vs. 4, *p* < 0.001).

**Table 2:** Subjective image quality.

	Original protocol Noise index 15 ASiR-V 90% *N* = 152 Median (Range)	Modified protocol Noise index 20 DLIR-H *N* = 155 Median (Range)	*P*-Value	Difference between methods Median (95% CI)	Non-inferiority margin	Superiority margin
Main/lobar pulmonary arteries						
Quality-rated by reader 1	4 (2; 5)	4 (3; 5)	< 0.001	0 (0; 0)*	> −0.2	> 0.2
Quality-rated by reader 2	4 (2; 5)	5 (3; 5)	< 0.001	1 (1; 1)**	> −0.2	> 0.2
Mean quality-rating by both readers	4 (2; 5)	4.5 (3; 5)	< 0.001	0.5 (0.5; 1)**	> −0.2	> 0.2
Segmental/subsegmental pulmonary arteries						
Quality-rated by reader 1	4 (2; 5)	4 (2; 5)	0.024	0 (0; 0)*	> −0.2	> 0.2
Quality-rated by reader 2	3 (1; 5)	4 (2; 5)	< 0.001	1 (1; 1)**	> −0.2	> 0.2
Mean quality-rating by both readers	3.5 (1.5; 4.5)	4 (2.5; 5)	< 0.001	0.5 (0.5; 0.5)**	> −0.2	> 0.2

p-values are from Wilcoxon rank-sum test; *modified protocol non-inferior **modified protocol superior.

**Fig. 4:**
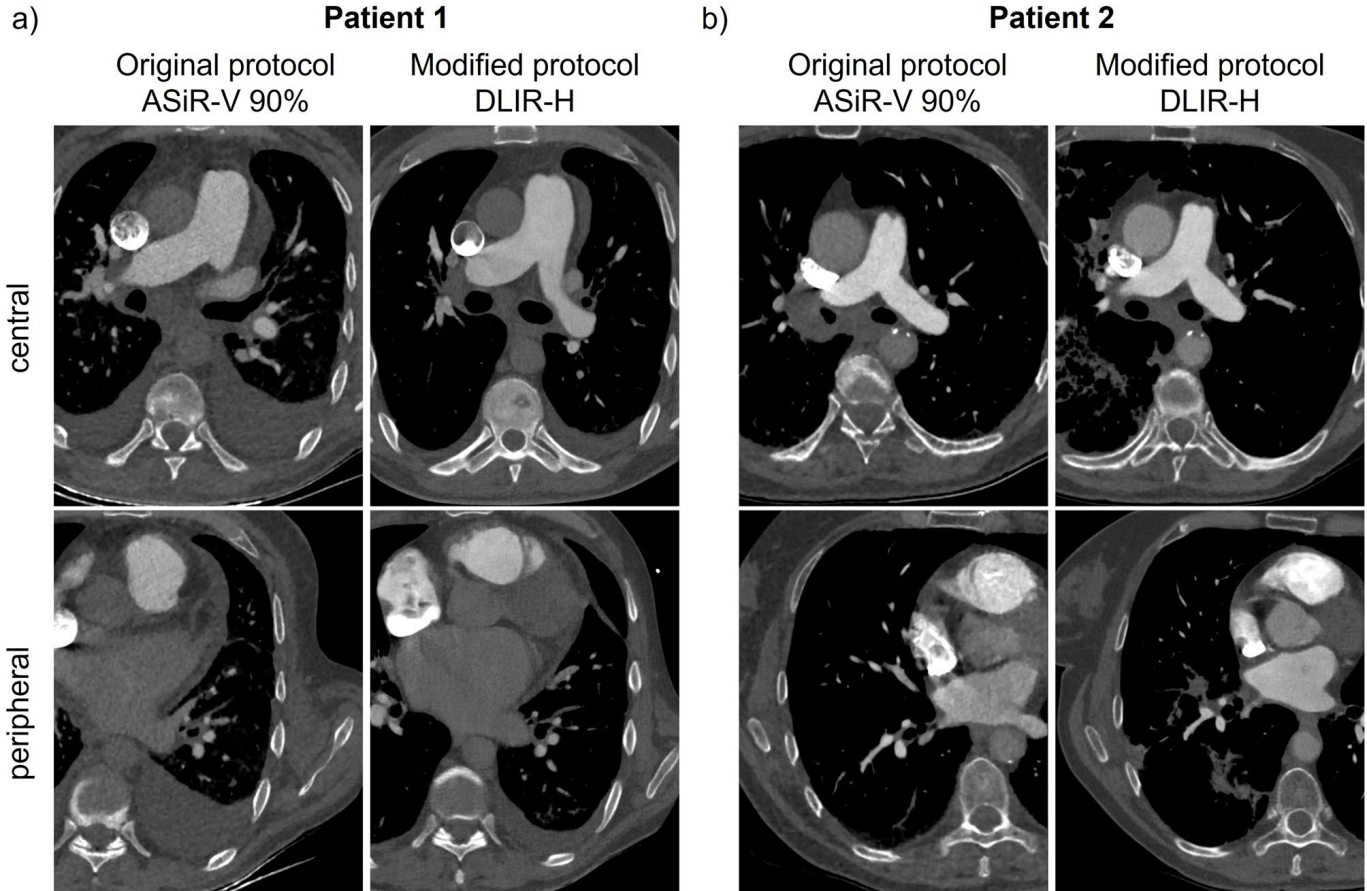
Comparison of image impression. Representative example figures of two patients who happened to undergo CTPA both before and after the protocol change are shown. Patient 1 is a 48-year old male and patient 2 is a 66-year old female. Representative images slices were selected showing the central and peripheral pulmonary arteries.

Subjective image quality for the central and lobar pulmonary arteries was confirmed as non-inferior for both readers. Superiority of the modified protocol was demonstrated for reader 2 and the average of both readers (Table 2). The same results were seen for the segmental pulmonary arteries with non-inferiority for both readers and superiority confirmed for reader 2 and for the average of both readers. Representative example figures of two patients who happened to undergo CTPA both before and after the protocol change can be found in Fig. 4.

The inter-reader analysis revealed a high agreement of both subjective image quality readers with a percent agreement of 0.94 (Confidence Interval (CI): 0.92;0.95) for the central artery and 0.95 (CI: 0.94;0.96) in the segmental arteries of the original protocol. In the modified protocol the inter-reader agreement was slightly lower with a percent agreement of 0.86 (CI: 0.84; 0.88) in the central artery and 0.91 (CI: 0.90;0.93) in the segmental arteries.

#### Subgroup analysis stratified by body size

**Fig. 5:**
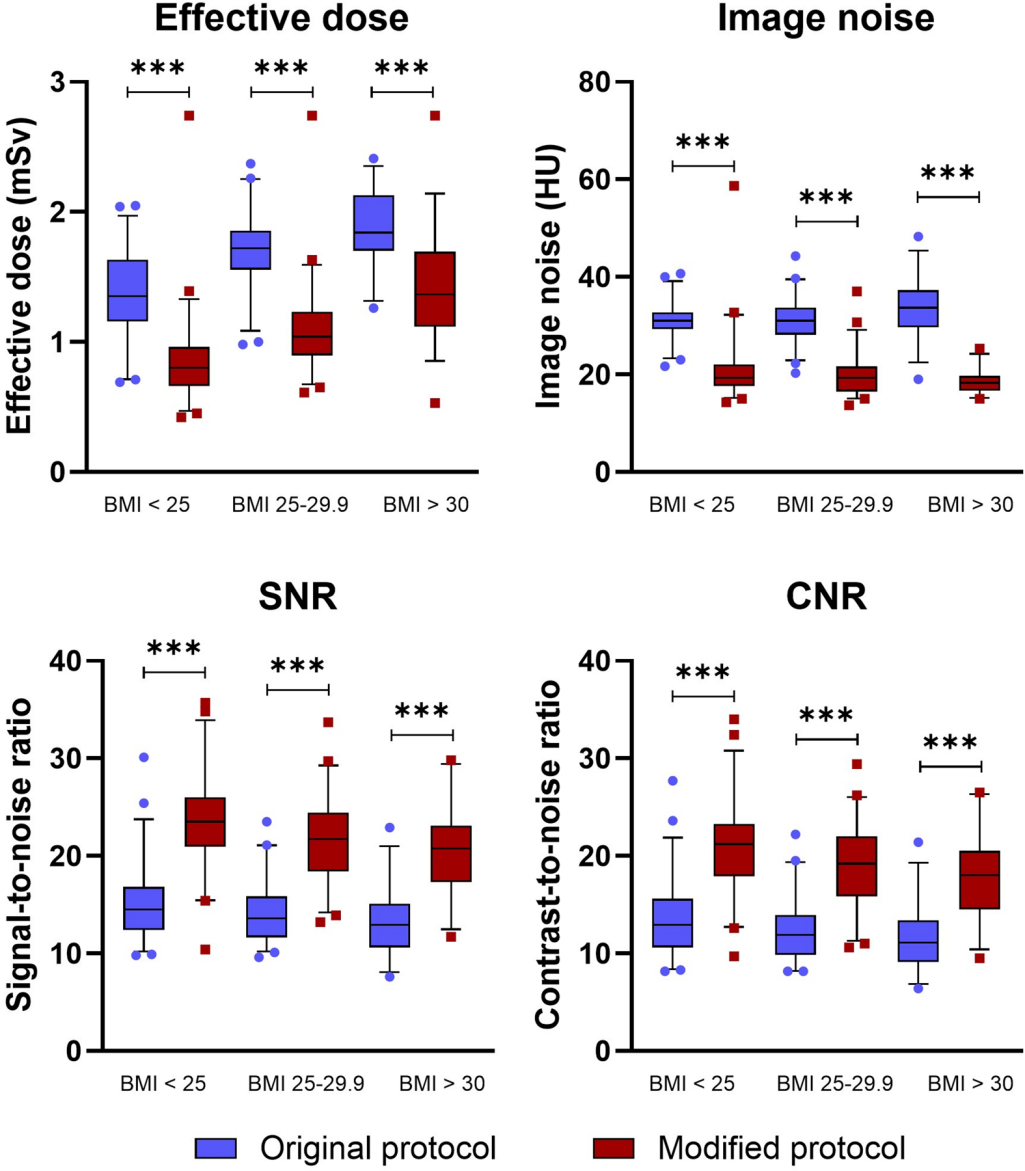
Effective dose and objective image quality for the main pulmonary artery stratified by body size. Effective dose calculated for both protocols. Objective Image quality was evaluated based on image noise, SNR and CNR. Data are presented as median and 5th–95th percentile. Blue boxplots indicate the original protocol (standard dose and ASiR-V 90%) and red boxplots the modified protocol (reduced dose and DLIR-H). Statistical significance was calculated by Mann-Whitney test and displayed as *** *p* < 0.001.

Median effective radiation dose decreased by 42% (1.35 vs. 0.78 mSv, *p* < 0.001) for normal-weight patients, by 40% (1.72 vs. 1.04 mSv, *p* < 0.001) for overweight patients and by 25% (1.84 vs. 1.38 mSv, *p* < 0.001) for obese patients (Fig. 5). Trends in objective image quality parameters were very consistent across body sizes. Detailed results of objective image quality parameters for each subgroupare visualized in Fig. 5 for central pulmonary arteries and in Supplementary Figure 7 for segmental pulmonary arteries and provided in Supplementary Table 5, Supplementary Table 6 and Supplementary Table 7. For all three subgroups, non-inferiority and superiority of the modified protocol were confirmed with regards to image noise, SNR and CNR of the main pulmonary artery (Supplementary Fig. 8a). For the segmental pulmonary arteries, non-inferiority could not be confirmed for most parameters in the subgroup analysis due to a lack of statistical power (Supplementary Fig. 8b).

#### PE-specific contrast-to-noise ratio for patients with confirmed PE

**Fig. 6:**
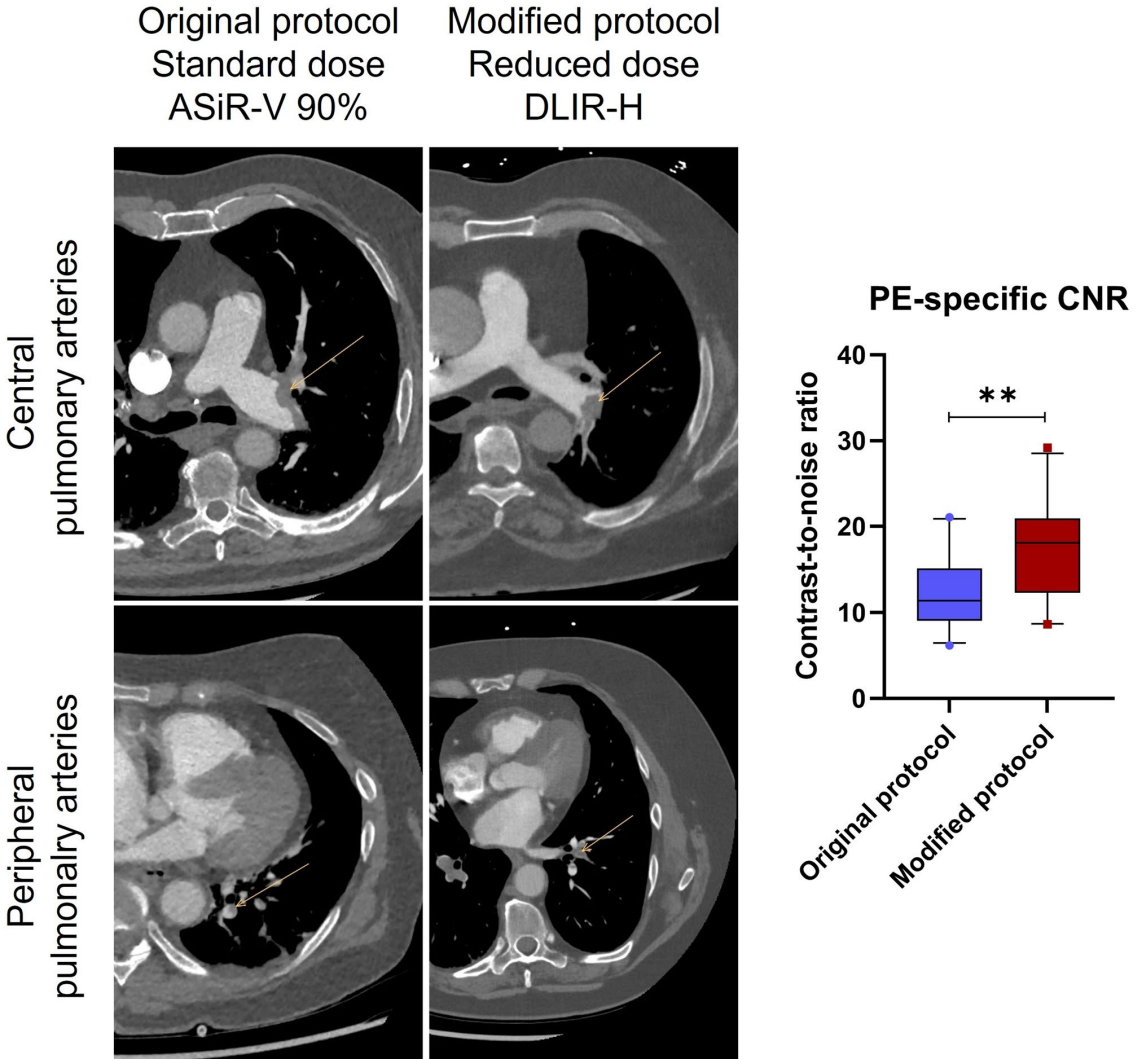
Subgroup analysis for patients with confirmed PE. (**a**) Representative example images of four different patients before and after the protocol change for the central and peripheral pulmonary artery. Arrows indicate pulmonary embolus. (**b**) Evaluation of the PE-specific CNR as subgroup analysis for patients with verified PE (*n* = 47, 25 within the original protocol, 22 within the modified protocol). The PE-specific contrast-to-noise ratio was quantified as intravascular attenuation adjacent to the pulmonary embolus minus the attenuation within the embolus, divided by intravascular image noise as described in^18^. Statistical significance was calculated by Mann-Whitney test and displayed as ** *p* < 0.01.

A total of 47 patients with confirmed PE (25 examined with the original protocol and 22 with the modified protocol) were analyzed for PE-specific CNR (contrast between pulmonary embolus and adjacent blood). Figure 6 shows the subgroup analysis of PE-specific CNR, comparing the original and modified protocols. A significantly higher PE-specific CNR was seen with the modified protocol compared to the original protocol (11.4 vs. 18.1, *p* < 0.01).

## Discussion

Our analysis shows that a modified CTPA protocol with reduced dose and high-strength DLIR (DLIR-H) is non-inferior in image quality compared to the original protocol using standard dose and high-strength iterative reconstruction (ASiR-V 90%). The analysis of objective and subjective image quality in patients with suspected PE revealed non-inferiority on all levels and even superiority of the modified protocol for the central pulmonary arteries. DLIR-H made it possible to increase the reference noise index and thus indirectly allowed for a substantial additional reduction in radiation dose lowering it to the sub-milliSievert range.

The potential of DLIR for additional improvement in image quality and/or radiation dose reduction has been investigated for various other applications of cardiovascular CT including cardiac CT^20–24^, CT of the aorta^25^, CT for planning of transcatheter aortic valve repair^26,27^ and head and neck CT angiography^28^.

Given the large number of CTPA scans performed for suspected PE and the long-term risks of radiation exposure, reducing radiation dose is particularly imperative for this commonly performed examination. In a previous study on DLIR in CTPA, imaging data from the same patient cohort were reconstructed with filtered back projection (FBP), adaptive statistical iterative reconstruction (ASiR-V 30%, 60% and 90%) and DLIR (low, medium and high strength). This previous study demonstrated significant improvements in objective image quality with DLIR compared to ASiR-V with the best results seen with DLIR-H^18^. This investigation suggested that DLIR-H may allow further dose reduction for patients with suspected PE but could not directly demonstrate this as no actual dose reduction had been implemented.

In the first part of our study, we performed a phantom study to estimate the level of additional radiation dose reduction enabled by DLIR-H compared to ASiR-V 90% using a medium and large thorax phantom body representing normal-weight and obese individuals. Non-inferior SNR was seen with DLIR-H even for substantial dose reductions of 49–71% compared to standard dose and ASIR-V 90% reconstruction. Based on the results of this phantom study, we modified our clinical protocol by switching to DLIR-H for reconstruction and reducing dose (by increasing the noise index from 15 to 20). In the clinical cohort study, this change in CTPA protocol translated into a substantial reduction in radiation dose by approximately 42% with a median effective dose of the modified protocol just below 1 mSv.

Only one previous study has directly compared a reduced-dose CTPA protocol with DLIR to a standard-dose protocol with iterative reconstruction. This previous study showed that DLIR improved the image quality while reducing the radiation dose in CTPA for suspected PE^19^. In this previous study, objective image quality measured as SNR and CNR was improved by 17–18% while radiation dose was decreased by 20–21%^19^. The improvement in image quality suggests that even large dose reduction would have been possible with non-inferior image quality. The results of this previous study cannot be directly compared to our results as their analysis used the iterative reconstruction and DLIR algorithms of a different vendor.

In our clinical study, the modified protocol using reduced dose and DLIR-H was non-inferior and even superior to the original protocol in image quality using standard dose and ASiR-V 90%. Despite a 42% dose reduction, SNR and CNR for the central pulmonary arteries were 64–66% higher in the modified protocol. For the segmental pulmonary arteries, there were no significant differences in SNR or CNR between the both protocols. However, also for the segmental pulmonary arteries, these metrics of image quality were confirmed to be non-inferior in image quality despite the substantial dose reduction.

Subjective image quality for both the central and the segmental/subsegmental pulmonary arteries was non-inferior and even superior with the modified protocol. As demonstrated in the phantom part of our manuscript, increasing noise index (decreasing dose) with the same reconstruction technique leads to increased image noise and reduced signal-to-noise ratio. Thus, the effect of preserved or even superior image quality at reduced dose can be attributed to DLIR. This improvement of subjective image quality goes beyond the published literature. At identical dose, Klemenz et al. demonstrated significantly higher image quality ratings for DLIR-M and DLIR-H compared to iterative reconstruction^18^. Lenfant et al. previously demonstrated a significant improvement of subjective image quality with moderate dose reduction (by 20–21%)^19^. It speaks to the capability of DLIR that our study observed improved subjective image quality despite a much more substantial (41%) dose reduction.

We performed a BMI-stratified subgroup analysis, which revealed that the results of our study were very consistent across the subgroups of normal-weight, overweight and obese patients. All subgroups of patients benefited from a significant reduction of radiation dose ranging from 42% for normal-weight patients to 25% for obese patients. This demonstrates a benefit of reduced-dose CTPA using DLIR in clinical practice for all patients independent of body size, even though the dose reduction was smaller and the segmental vessel results were less robust in obese patients compared to normal-weight patients. Compared to our phantom study, the dose reduction in obese patients was higher. This effect can be explained, since the large thorax phantom only mimics a higher BMI by expanding the thorax diameter, whereas the body composition and the thorax diameter of obese patients might not be consistent with the diameter of the obese phantom extension ring.

We applied a 5% non-inferiority margin for image noise based on our clinical (radiological) judgement that a difference of this magnitude would not be clinically relevant. The same 5% threshold (corresponding to a 0.2 point difference) was applied to subjective image quality scores. There is no accepted consensus in the literature how non-inferiority margins for studies on CT image quality should be defined. For subjective image quality ratings, a 0.2 point non-inferiority margin has been used previously^29^, while others have used a 0.5 point difference as a more lenient NI margin^30,31^. Therefore, the non-inferiority margin used in our study can be considered relatively conservative and unlikely to miss any clinically relevant differences.

Our study has several limitations including the retrospective and non-randomized study design. Unmeasured confounders across cohorts might have affected the results. Furthermore, this study was designed as a single-center study with CT hardware and reconstruction algorithms from one manufacturer. The diagnostic accuracy regarding PE detection was not analyzed.

Since the chronicity of the PE was not determined, this may have influenced the CNR findings due to varying embolus density between both clinical cohorts. In the future, multi-center studies with many different CT systems and DLIR algorithms of various vendors in combination with fully automated PE detection software may increase the generalizability of our results.

In conclusion, our study provides direct evidence that DLIR allows radiation dose for CTPA to be reduced by an additional 41% with non-inferior objective and subjective image quality compared to state-of-the-art iterative reconstruction.

## Methods

### Phantom study

#### Phantom setup

An anthropomorphic thorax phantom (QRM-Thorax, QRM GmbH, Möhrendorf) was used to determine the extent of radiation dose reduction. The phantom was scanned both with a medium (350 × 250 mm) and a large (400 × 300 mm) tissue-equivalent extension ring (QRM GmbH, Möhrendorf) to represent non-obese and obese patients, respectively. The contrast agent tubes were centrally embedded in an agar-agar inlay and prepared using 15 ml and 50 ml Falcon tubes, each filled with iodinated contrast agent at varying concentrations (10 mg/ml, 12.5 mg/ml, 15 mg/ml, 17.5 mg/ml, and 20 mg/ml), to achieve a target CT density of 350–450 HU (Fig. 1a).

#### Image acquisition and reconstruction

The phantom study was conducted on a 256-detector-row CT system (Revolution Apex, GE HealthCare). A fixed tube voltage of 100 kVp and a rotation time of 0.28 s were used. The dose is varied by adjusting the noise index (NI), which describes the expected standard deviation of Hounsfield Units (HU) in a well-defined phantom, for a given reconstruction algorithm and slice thickness. According to our in-house protocol, the NI are defined based on a reference slice thickness of 2.5 mm. The NI for the CT protocol was incrementally increased from 15 to 25. Images were reconstructed using hybrid iterative reconstruction ASiR-V (GE HealthCare) at 90% strength and DLIR (TrueFidelity™, GE HealthCare) in high strength (DLIR-H) with a slice thickness of 0.625 mm.

#### Image analysis

For the image analysis, the central tube (iodine concentration 10 mg/ml) was chosen as its larger diameter allowed more accurate measurements. For each dose level and each reconstruction, a circular region of interest (diameter: 25 pixels for phantom M; 20 pixels for phantom L) was placed within the tube on each slice. CT density and standard deviation of CT density were recorded. The results measured on 5 slices were averaged. The standard deviation of CT density was used as a metric of image noise. Signal-to-noise-ratio was calculated as CT density divided by the standard deviation of CT density.

### Clinical study

#### Patient selection and study design

Based on the results of our phantom experiments, we modified our clinical CTPA protocol in August 2024 to use DLIR for image reconstruction and simultaneously lowered the dose (by increasing the reference noise index from 15 to 20). After this clinically driven change in the CTPA protocol we retrospectively analyzed 307 consecutive patients who were examined with CTPA before (April through August 2024, *n* = 152) and after the change in protocol (August through November 2024, *n* = 155). No patient was excluded from the analysis to avoid selection bias. This retrospective study was performed as a single-center cohort study. Patients’ age, weight and height as well as radiation metrics were retrospectively collected.

#### Sample size calculation

Sample size was calculated for a non-inferiority study design with unpaired groups^32^. Based on a preliminary analysis of our cohort examined with the original protocol, we expected a mean noise in the control group (original protocol) of 32 HU with a standard deviation of 5.6 HU and a subjective image quality score of 4.0 with a standard deviation of 0.57. We defined the non-inferiority margin as a delta of no more than 5% from the original protocol and the superiority threshold as an improvement of more than 5% from the original protocol (1.6 HU for image noise and 0.2 for subjective image quality score). These margins were based on our clinical (radiological) judgement that a 5% difference in image noise would not be clinically relevant. For consistency, we applied the same 5% threshold to the subjective image quality ratings. With these assumptions, a sample size of *n* = 303 was calculated to be necessary to demonstrate non-inferiority with a 5% one-sided type I error and 80% statistical power.

#### Ethical approval and informed consent

The study was approved by the responsible Institutional Review Board (Ethical committee, Rostock University Medical Center) with waiver of informed consent and was conducted in accordance with the Declaration of Helsinki.

#### Image acquisition and reconstruction

All patients were examined on a 256-detector-row CT system (Revolution Apex, GE HealthCare). Acquisition parameters are shown in Table 3. Except for the reference noise index of (15 in the original protocol vs. 20 in the modified protocol) and the image reconstruction technique (ASiR-V 90% in the original protocol vs. DLIR-H in the modified protocol), all other acquisition parameters including the contrast protocol were kept equal. All images were reconstructed with a slice thickness of 0.625 mm.

**Table 3:** CT acquisition parameters for the patient study.

CT Parameter	Value	
Acquisition parameter		
Tube voltage [kV]	100	
Gantry rotation time [s]	0.28	
Tube current	Attenuation-based tube current modulation	
Noise index	Original protocol: 15	Modified protocol: 20
Image reconstruction	Original protocol: ASiR-V 90%	Modified protocol: DLIR-H
Contrast protocol		
Contrast volume [ml]	60–70	
Contrast concentration [mg/ml]	370–400	
Flow rate [ml/s]	4.0	
Saline chaser [ml]	40 at 4 ml/s	

#### Analysis of radiation exposure

The volumetric CT dose index (CTDI_vol_) and dose length product (DLP) were collected from the dose reports in the picture archiving and communication system. The effective dose was calculated by multiplying the DLP with a standard conversion factor for adult chest CT of 0.0146 mSv/mGy*cm^33^.

#### Analysis of objective image quality

Objective image quality was compared between the original protocol (original dose and ASiR-V 90%) and the modified protocol (reduced dose and DLIR-H).

For this purpose, circular regions of interest (ROIs) were positioned in the main pulmonary artery (diameter 20 pixels), a segmental pulmonary artery (diameter 7 pixels) and the paraspinal muscle (diameter 10 pixels) with subsequent determination of the image noise, signal-to-noise ratio (SNR) and contrast-to-noise ratio (CNR) as described in^18^. For the subset of patients with confirmed PE, we additionally quantified the PE-specific contrast-to-noise ratio as the CNR between embolus and adjacent blood in the vessel lumen^18^. To ensure valid measurements of CT attenuation and noise, we included only cases of PE where a circular ROI with a diameter of at least 3 pixels could be placed within the embolus. The ROI within the adjacent blood vessel was set as large as possible within the vessel lumen, with a minimum diameter of 5 pixels and averaged over measurements in three adjacent slices in the same vessel.

#### Analysis of subjective image quality

Assessment of subjective image quality was carried out by two radiologists, who were blinded to the acquisition protocol and independently assessed image quality for diagnosing or excluding PE using a 5-point scale ranging from 5 (excellent image quality allowing unambiguous diagnosis of the presence or absence of a clot) to 1 (non-diagnostic image quality, PE can neither be confirmed nor ruled out). Ratings were carried out for the main pulmonary arteries and the segmental pulmonary arteries separately.

#### Statistical analysis

Clinical and imaging data are presented as median and minimum-maximum-range. Statistical differences were calculated by Wilcoxon rank-sum (Mann–Whitney) test for non-normally distributed continuous data and chi-square test for categorical data. A p-value < 0.05 was considered as statistically significant. For inter-reader agreement the quadratic weighted percent agreement was calculated with 95% confidence interval. For non-inferiority and superiority testing, the median difference between methods along with its 95% confidence interval (CI) were calculated and compared to predefined margins. Non-inferiority of the modified protocol was confirmed if the 95% CI did not cross the pre-specified non-inferiority margin (i.e., the interval lay entirely outside the inferiority range). Superiority was established if the observed difference between methods indicated an improvement and the 95% CI did not cross the pre-specified superiority margin (i.e., the interval lay entirely outside the non-superiority range). Subgroup analyses were performed based on body size stratification: normal weight (BMI < 25 kg/m²), overweight (25.0–29.9 kg/m²) and obese (≥ 30 kg/m²). Stata 18.0 (Stata Corporation, College Station, TX, U.S.A.) and Graphpad Prism (Version 10.4.1, Graphpad Software LLC) were used for statistical analyses and illustration.

## Supplementary Information

Below is the link to the electronic supplementary material.

Supplementary Figure 1

Supplementary Figure 2

Supplementary Table 3

Supplementary Table 4

Supplementary Table 5

Supplementary Table 6

Supplementary Figure 7

Supplementary Figure 8

## Data Availability

The data that support the findings of this study are available from the corresponding author upon reasonable request.

## Abbreviations

ASiR: V Adaptive statistical iterative reconstruction
CI: Confidence Interval
CNR: Contrast-to-noise ratio
CT: Computed tomography
CTA: Computed tomography angiography
CTDI_vol_: Volume computed tomography dose index
CTPA: Computed tomography pulmonary angiography
DLIR: Deep learning-based image reconstruction
DLP: Dose length product
FBP: Filtered back projection
HU: Hounsfield units
NI: Noise index
PE: Pulmonary embolism
ROI: Region of interest
SNR: Signal-to-noise ratio
