# Supplementary figures and images for: Deep-learning based image reconstruction enables reduced dose CT pulmonary angiography with non-inferior image quality
Source: Sci Rep. 2026 Jun 9;16:17849. doi: 10.1038/s41598-026-56545-y (PMC13250109; doi:10.1038/s41598-026-56545-y)

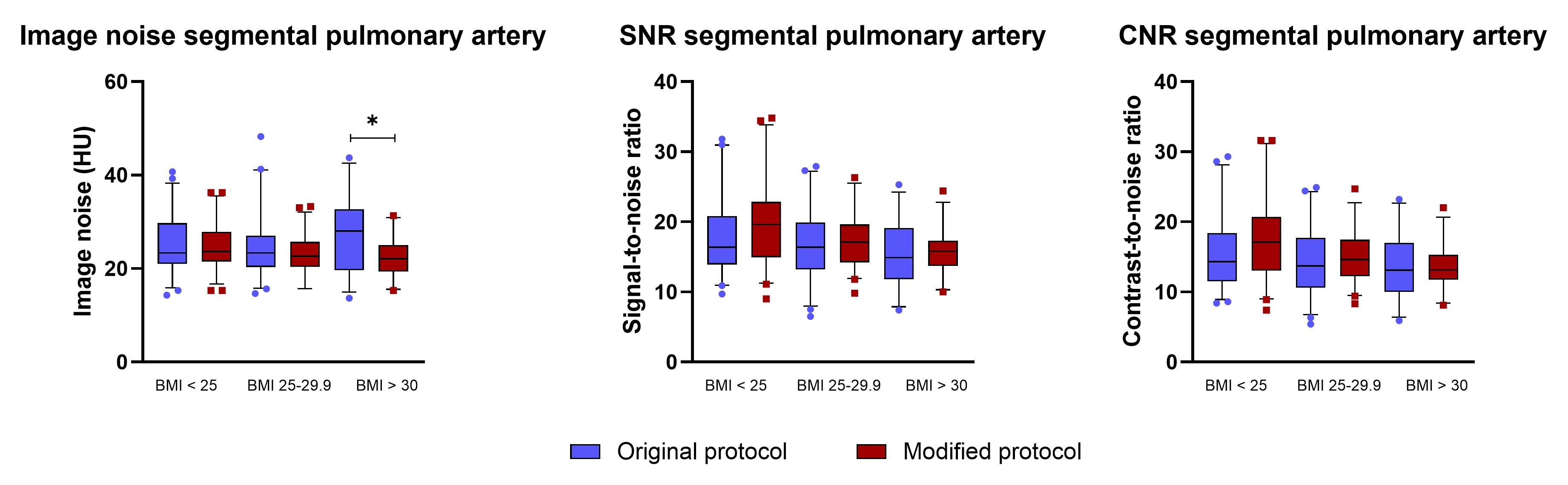

Supplement: Supplementary file 1 — Supplementary Figure 1 [file 41598_2026_56545_MOESM1_ESM.jpg]

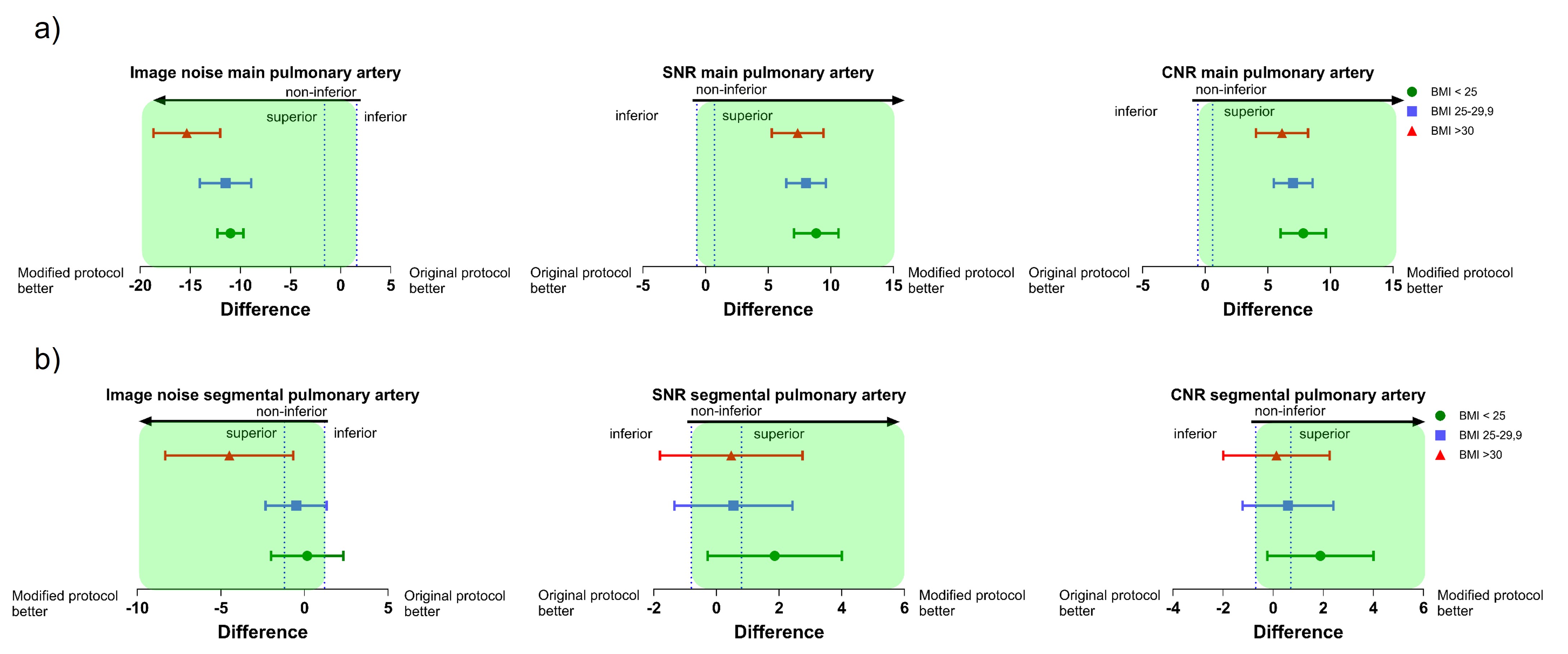

Supplement: Supplementary file 2 — Supplementary Figure 2 [file 41598_2026_56545_MOESM2_ESM.jpg]
