# Supplementary material for: Deep-learning based image reconstruction enables reduced dose CT pulmonary angiography with non-inferior image quality
Source: Sci Rep. 2026 Jun 9;16:17849. doi: 10.1038/s41598-026-56545-y (PMC13250109; doi:10.1038/s41598-026-56545-y)
Supplement: Supplementary file 3 — Supplementary Table 3 [file 41598_2026_56545_MOESM3_ESM.docx]

**Supplementary Table 4: Objective image quality**

|  | **Original protocol**  **Noise index 15**  **ASiR V 90%**  **N=152**  **Median (Range)** | **Modified protocol**  **Noise index 20**  **DLIR- H**  **N=155**  **Median (Range)** | **P-Value** | **Difference between methods**  **Median (95% CI)** | **Non-inferiority margin** | **Superiority margin** |
| --- | --- | --- | --- | --- | --- | --- |
| Attenuation paraspinal muscle [HU] | 52 (24.3; 74.7) | 52.7 (37.3; 73.3) | 0.901 | 0 (-2; 2) |  |  |
| **Main pulmonary artery** |  |  |  |  |  |  |
| Intravascular attenuation [HU] | 421 (253; 788) | 438 (252; 988) | 0.523 | -9 (-36.0; 18.3) | > -21 | > 21 |
| Intravascular image noise [HU] | 31.7 (19; 52) | 19.3 (13.7; 58.7) | <0.001 | -12.3 (-13.3; -11.3)** | < 1.6 | < -1.6 |
| Signal-to-Noise Ratio | 13.6 (7.6; 30.1) | 22.3 (10.4; 35.7) | <0.001 | 8.23 (7.32; 9.20)** | > -0.7 | > 0.7 |
| Contrast-to-Noise Ratio | 11.8 (6.4; 27.7) | 19.6 (9.5; 34) | <0.001 | 7.20 (6.25, 8.18)** | > - 0.6 | > 0.6 |
| **Segmental pulmonary artery** |  |  |  |  |  |  |
| Intravascular attenuation [HU] | 381 (251; 788) | 403 (253; 928) | 0.525 | 7.67 (-15.0; 31.3)* | > -19 | > 19 |
| Intravascular image noise [HU] | 23.8 (12.7; 48.3) | 23.7 (15.3; 36.3) | 0.239 | -0.67 (-2.0, 0.67)* | < 1.2 | <-1.2 |
| Signal-to-Noise Ratio | 16.4 (6.5; 31.8) | 16.8 (9; 34.8) | 0.205 | 0.70 (-0.39, 1.76)* | > -0.8 | > 0.8 |
| Contrast-to-Noise Ratio | 14.3 (5.4; 29.3) | 14.6 (7.4; 31.6) | 0.209 | 0.65 (-0.38, 1.68)* | > -0.7 | > 0.7 |

*p-values are from Wilcoxon rank-sum test; *modified protocol non-inferior **modified protocol superior*
