# Supplementary material for: Deep-learning based image reconstruction enables reduced dose CT pulmonary angiography with non-inferior image quality
Source: Sci Rep. 2026 Jun 9;16:17849. doi: 10.1038/s41598-026-56545-y (PMC13250109; doi:10.1038/s41598-026-56545-y)
Supplement: Supplementary file 4 — Supplementary Table 4 [file 41598_2026_56545_MOESM4_ESM.docx]

**Supplementary Table 5: Subgroup analysis based on body size: normal-weight patients.**

| **Normal-weight patients**  **BMI <25 kg/m^2^** | **Original protocol**  **Noise index 15**  **ASiR-V 90%**  **N=47**  **Median (Range)** | **Modified protocol**  **Noise index 20**  **DLIR- H**  **N=49**  **Median (Range)** | **P-Value** | **Difference between methods**  **Median (95% CI)** | **Non-inferiority margin** | **Superiority margin** |
| --- | --- | --- | --- | --- | --- | --- |
| Attenuation paraspinal muscle [HU] | 52.0 (41.0; 74.7) | 54.3 (39.3; 73.3) | 0.479 | 1 (1.67; 4) |  |  |
| **Main pulmonary artery** |  |  |  |  |  |  |
| Intravascular attenuation [HU] | 432 (279; 760) | 482 (252; 988) | 0.312 | 27.7 (-30.3; 81.3) | > -21 | > 21 |
| Intravascular image noise [HU] | 31 (21.7; 40.7) | 19.3 (14.3; 58.7) | <0.001 | -11 (-12.3; -9.7)** | < 1.6 | < -1.6 |
| Signal-to-Noise Ratio | 14.5 (9.8; 30.1) | 23.5 (10.4; 35.7) | <0.001 | 8.97 (7.06; 10.6)** | > -0.7 | > 0.7 |
| Contrast-to-Noise Ratio | 12.9 (8.2; 27.7) | 21.2 (9.7; 34) | <0.001 | 7.96 (6.02; 9.63)** | > - 0.6 | > 0.6 |
| **Segmental pulmonary artery** |  |  |  |  |  |  |
| Intravascular attenuation [HU] | 394 (257; 723) | 450 (256; 928) | 0.045 | 49.7 (1; 88)* | > -19 | > 19 |
| Intravascular image noise [HU] | 23.3 (14.3; 40.7) | 23.7 (15.3; 36.3) | 0.781 | 0.33 (-2, 2.33) | < 1.2 | <-1.2 |
| Signal-to-Noise Ratio | 16.4 (9.7; 31.8) | 19.6 (9; 34.8) | 0.083 | 1.71 (-0.28; 4.00)* | > -0.8 | > 0.8 |
| Contrast-to-Noise Ratio | 14.3 (8.4; 29.3) | 17.1 (7.4; 31.6) | 0.082 | 1.84 (-0.23; 4.00)* | > -0.7 | > 0.7 |

*Objective image quality parameters are shown for the original protocol (standard dose, ASiR-V 90%) and the modified protocol reduced dose, DLIR-H) for patients with BMI < 25* kg/m^2^. *P-values are from Wilcoxon rank-sum test; *modified protocol non-inferior **modified protocol superior*
