# Supplementary material for: Deep-learning based image reconstruction enables reduced dose CT pulmonary angiography with non-inferior image quality
Source: Sci Rep. 2026 Jun 9;16:17849. doi: 10.1038/s41598-026-56545-y (PMC13250109; doi:10.1038/s41598-026-56545-y)
Supplement: Supplementary file 5 — Supplementary Table 5 [file 41598_2026_56545_MOESM5_ESM.docx]

**Supplementary Table 6: Subgroup analysis based on body size: overweight patients.**

| **Overweight patients**  **BMI 25.0-29.9 kg/m^2^** | **Original protocol**  **Noise index 15**  **ASiR-V 90%**  **N=46**  **Median (Range)** | **Modified protocol**  **Noise index 20**  **DLIR- H**  **N=46**  **Median (Range)** | **P-Value** | **Difference between methods**  **Median (95% CI)** | **Non-inferiority margin** | **Superiority margin** | |
| --- | --- | --- | --- | --- | --- | --- | --- |
| Attenuation paraspinal muscle [HU] | 52.8 (24.3; 71) | 52.5 (38.7; 66) | 0.981 | 0 (-3.33, 3.67) |  |  | |
| **Main pulmonary artery** |  |  |  |  |  |  | |
| Intravascular attenuation [HU] | 409 (253; 768) | 431 (264; 687) | 0.907 | -1.5 (-47; 46) | > -21 | > 21 | |
| Intravascular image noise [HU] | 31 (20.3; 44.3) | 19.2 (13.7; 37) | <0.001 | -11.7 (-13.3, -9.67)** | < 1.6 | < -1.6 | |
| Signal-to-Noise Ratio | 13.6 (9.6; 23.5) | 21.9 (13.2; 33.7) | <0.001 | 7.99 (6.44; 9.60)** | > -0.7 | > 0.7 | |
| Contrast-to-Noise Ratio | 11.8 (8.2; 22.2) | 19.2 (10.6; 29.4) | <0.001 | 6.94 (5.47, 8.57)** | > - 0.6 | > 0.6 | |
| **Segmental pulmonary artery** |  |  |  |  |  |  | |
| Intravascular attenuation [HU] | 369 (260; 788) | 371 (268; 639) | 0.519 | 13.8 (-23.3, 47.3) | > -19 | > 19 | |
| Intravascular image noise [HU] | 23.3 (14.7; 48.3) | 22.7 (15.7; 33.3) | 0.482 | -0.66 (-2.33, 1.33) | < 1.2 | <-1.2 | |
| Signal-to-Noise Ratio | 16.6 (6.5; 27.9) | 16.9 (9.8; 26.3) | 0.548 | 0.54 (-1.34, 2.43) | > -0.8 | > 0.8 | |
| Contrast-to-Noise Ratio | 14 (5.4; 24.9) | 14.5 (8.3; 24.7) | 0.497 | 0.54 (-1.22, 2.40) | > -0.7 | > 0.7 |  |

*Objective image quality parameters are shown for the original protocol (standard dose, ASiR-V 90%) and the modified protocol reduced dose, DLIR-H) for patients with BMI 25.0-29.9 kg/m^2^. P-values are from Wilcoxon rank-sum test; *modified protocol non-inferior **modified protocol superior*
