# Supplementary material for: Deep-learning based image reconstruction enables reduced dose CT pulmonary angiography with non-inferior image quality
Source: Sci Rep. 2026 Jun 9;16:17849. doi: 10.1038/s41598-026-56545-y (PMC13250109; doi:10.1038/s41598-026-56545-y)
Supplement: Supplementary file 6 — Supplementary Table 6 [file 41598_2026_56545_MOESM6_ESM.docx]

**Supplementary Table 7: Subgroup analysis based on body size: obese patients.**

| **Obese patients**  **BMI ≥ 30 kg/m^2^** | **Original protocol**  **Noise index 15**  **ASiR-V 90%**  **N=35**  **Median (Range)** | **Modified protocol**  **Noise index 20**  **DLIR- H**  **N=32**  **Median (Range)** | **P-Value** | **Difference between methods**  **Median (95% CI)** | **Non-inferiority margin** | **Superiority margin** |
| --- | --- | --- | --- | --- | --- | --- |
| Attenuation paraspinal muscle [HU] | 50 (26; 68.7) | 50.8 (37.7; 64.7) | 0.940 | 0 (-4.33, 3.67) |  |  |
| **Main pulmonary artery** |  |  |  |  |  |  |
| Intravascular attenuation [HU] | 406 (262; 717) | 376 (252; 593) | 0.051 | -52 (-111; 0.33) | > -21 | > 21 |
| Intravascular image noise [HU] | 33.7 (19; 48.3) | 18.3 (15; 25.3) | <0.001 | -15.3 (-17.7; -13)** | < 1.6 | < -1.6 |
| Signal-to-Noise Ratio | 12.9 (7.6; 22.9) | 20.8 (11.7; 29.8) | <0.001 | 7.39 (5.28; 9.41)** | > -0.7 | > 0.7 |
| Contrast-to-Noise Ratio | 11.1 (6.4; 21.4) | 18 (9.5; 26.5) | <0.001 | 6.26 (4.05; 8.21)** | > - 0.6 | > 0.6 |
| **Segmental pulmonary artery** |  |  |  |  |  |  |
| Intravascular attenuation [HU] | 373 (251; 721) | 329 (255; 542) | 0.127 | -30.5 (-83; 12.3) | > -19 | > 19 |
| Intravascular image noise [HU] | 28 (13.7; 43.7) | 22.2 (15.3; 31.3) | 0.020 | -5 (-8.33; -0.67)* | < 1.2 | <-1.2 |
| Signal-to-Noise Ratio | 14.9 (7.4; 25.3) | 15.8 (10; 24.4) | 0.581 | 0.71 (-1.80; 2.75) | > -0.8 | > 0.8 |
| Contrast-to-Noise Ratio | 13.1 (5.9; 23.2) | 13.1 (8.1; 22) | 0.812 | 0.23 (-1.99; 2.25) | > -0.7 | > 0.7 |

*Objective image quality parameters are shown for the original protocol (standard dose, ASiR-V 90%) and the modified protocol reduced dose, DLIR-H) for patients with BMI ≥ 30 kg/m^2^. P-values are from Wilcoxon rank-sum test; *modified protocol non-inferior **modified protocol superior*
